# Crystal structures of three homologues with increasing ring size: 2-meth­oxy-4-(thio­phen-2-yl)-5,6,7,8-tetra­hydro­quinoline-3-carbo­nitrile, 2-meth­oxy-4-(thio­phen-2-yl)-6,7,8,9-tetra­hydro-5*H*-cyclo­hepta­[*b*]pyridine-3-carbo­nitrile and 2-meth­oxy-4-(thio­phen-2-yl)-5,6,7,8,9,10-hexa­hydrocyclo­octa[*b*]pyridine-3-carbo­nitrile

**DOI:** 10.1107/S2056989023001883

**Published:** 2023-03-15

**Authors:** Ali M. S. Hebishy, Galal H. Elgemeie, Lobna M. Gouda, Peter G. Jones

**Affiliations:** a Chemistry Department, Faculty of Science, Helwan University, Cairo, Egypt; b Institut für Anorganische und Analytische Chemie, Technische Universität Braunschweig, Hagenring 30, D-38106 Braunschweig, Germany; Universität Greifswald, Germany

**Keywords:** crystal structure, pyridine, thio­phene, nitrile, hydrogen bond

## Abstract

The title compounds, C_15_H_14_N_2_OS (**1a**), C_16_H_16_N_2_OS (**1b**), and C_17_H_18_N_2_OS (**1c**), form a homologous series in which the size of the saturated ring increases from six- to eight-membered (with four, five and six methyl­ene groups respectively). For **1b** and **1c**, the central (CH_2_)_*n*_ moieties are all displaced to the same side of their ring, and the CH_2_—CH_2_—CH_2_ angles are much wider than the standard *sp* ^3^ value; a database search indicates that these are general features of such ring systems. For **1a**, the thio­phene ring lies with the sulfur atom on the opposite side of the C_thio­phene_—C_pyridine_ bond to the cyano group, in contrast to **1b** and **1c**. For each compound, the packing may be described in terms of two ‘weak’ C—H⋯N hydrogen bonds, which link the mol­ecules to form one-dimensional (**1a**, **1c**) or three-dimensional (**1b**) assemblies.

## Abstract 2

The title compounds form a homologous but non-isotypic series with appreciable differences in mol­ecular form. In each case, the packing is determined by two C—H⋯N hydrogen bonds.

## 1. Chemical context

Recently, we started a widespread study of pyridones and related compounds and have described the synthesis of new *N*-substituted amino-2-pyridones (Azzam *et al.*, 2017*a* ▸,*b* ▸, 2020*a* ▸,*b* ▸,*c* ▸; see also Bolduc *et al.*, 2022 ▸). The synthetic applications of unsaturated nitriles as reaction inter­mediates for the preparation of a wide range of heterocyclic compounds has stimulated considerable inter­est in our group over the last decade (Khedr *et al.*, 2022*a* ▸,*b* ▸; Abdallah & Elgemeie, 2022 ▸). Since pyridines and their fused heterocycles have been shown to constitute a new class of anti­metabolites (De *et al.*, 2022 ▸), it is of inter­est to evaluate synthetic methods for the preparation of their analogues and demonstrate the effects of structural modifications on their biological activity (Elgemeie & Mohamed-Ezzat, 2022*a* ▸,*b* ▸). Many 2-meth­oxy­pyridine derivatives have previously been shown to possess anti­tubercular and anti­bacterial activities (Bodige *et al.*, 2019 ▸).

Some time ago we reported the synthesis of the condensed 2-meth­oxy-4-thienyl-3-cyano­pyridines (**1a**–**c**) *via* the reaction of cyclo­alkanones with 2-(2-thienyl­methyl­ene)malono­nitrile in refluxing methano­lic sodium hydroxide (Elgemeie *et al.*, 1991 ▸); we also presented experimental data and a proposed mechanism. In 2015, another research group repeated our reaction and synthesized **1c** using LiOEt instead of NaOEt (Maharani & Kumar, 2015 ▸). Here we are able to present the mol­ecular structures of **1a**–**c** determined with single crystal XRD. 
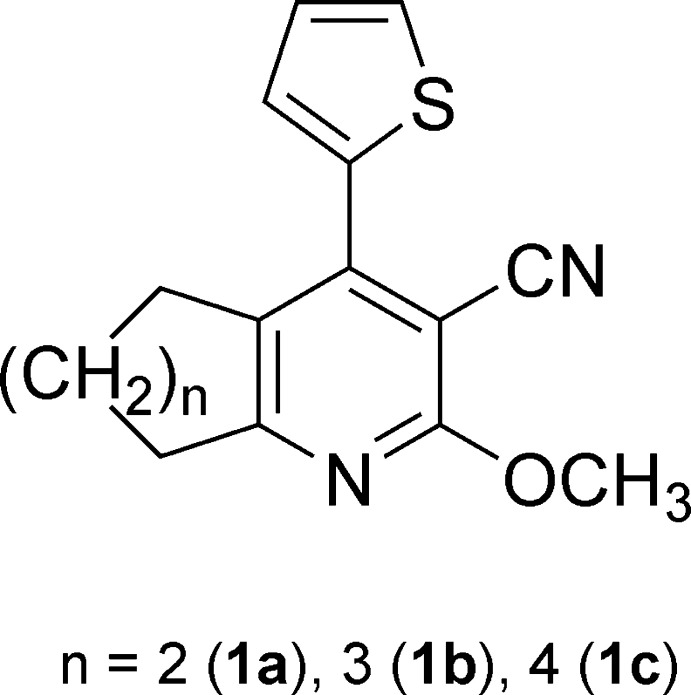


## 2. Structural commentary

**Figure 1:**
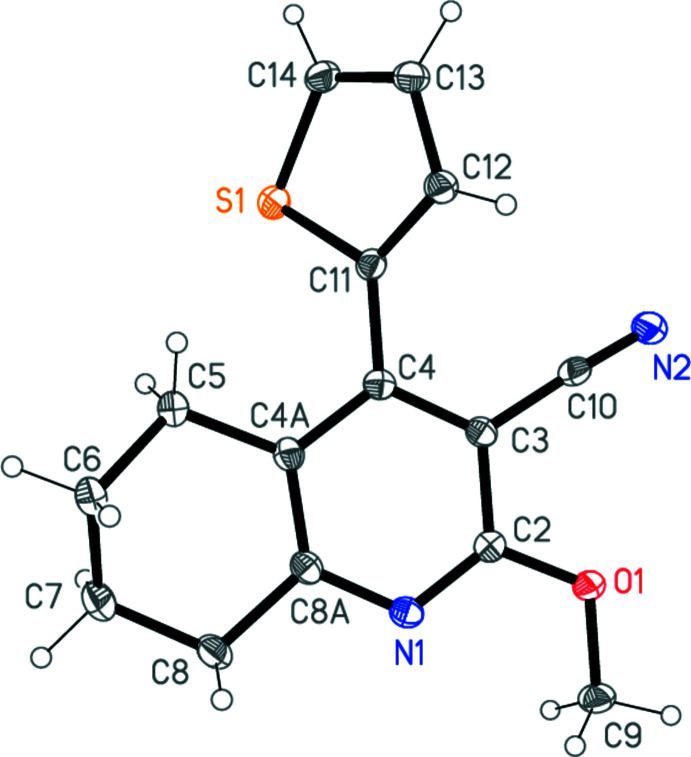
The mol­ecule of **1a** in the crystal. Ellipsoids represent 50% probability levels.

The structure determinations confirm the nature of the products **1a**–**c**. The three mol­ecules, which form a homologous series with increasing ring size, are shown in Figs. 1 ▸–3 ▸ ▸. The compounds all crystallize in space group *P*2_1_/*c* (or its equivalent *P*2_1_/*n*) but none of them is isotypic to any other. Bond lengths and angles may be considered normal for these compound types. For instance: the exocyclic angles N—C—C at the ring junctions are appreciably less than 120° and the CH_2_—CH_2_—CH_2_ angles of **1b** and **1c** are markedly wider than the standard value of 109.5° (see Tables 1 ▸–3 ▸ ▸). The overall form of the mol­ecules, however, differs between **1a** and the similar pair **1b**/**1c**.

For convenience, the rings are designated as follows: Ring *A*, thio­phene; ring *B*, pyridine-type ring; ring *C*, the ring containing the (CH_2_)_*n*_ moieties (as defined in the scheme, *e.g*. C4*A*,C5–C8,C8*A* for **1a**). The minor disorder components (see Section 6) are not considered. Tables 1 ▸–3 ▸ ▸ show the torsion angles of the rings *C*.

For **1a**, ring *C* displays a standard half-chair conformation, with C6 and C7 lying 0.481 (2) and 0.293 (2) Å, respectively, in opposite directions out of the plane defined by C5, C4*A*, C8*A* and C8. The thio­phene ring lies with the sulfur atom on the opposite side of the C4—C11 bond to the cyano group. The inter­planar angle between rings *A* and *B* is 45.33 (4)°.

**Figure 4:**
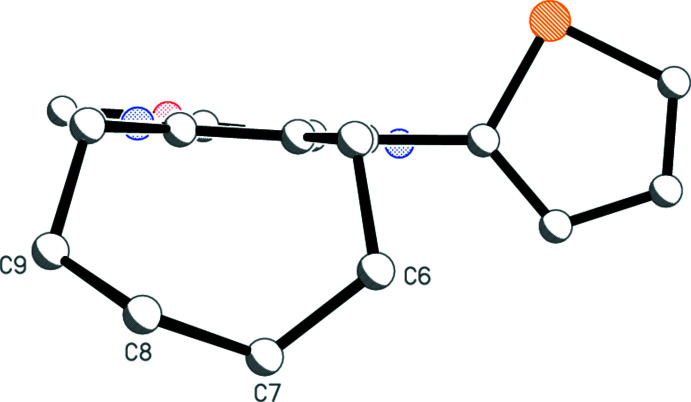
Side view of **1c** (radii arbitrary, H atoms omitted). The labelled atoms are displaced from the plane of the pyridine-type ring *B* (for definition, see text) by 1.343 (2), 2.360 (2), 1.913 (2) and 1.395 (2) Å, respectively.

For **1b** and **1c**, however, the thio­phene rings are differently positioned, with the sulfur atom on the same side of the C4—C12 (**1b**) or C4—C13 bond (**1c**) as the cyano group. The respective S1⋯N2 distances are 3.676 (1) and 4.070 (1) Å, too long to be considered significant inter­actions, and the inter­planar angles *A*/*B* are 61.40 (5) and 79.67 (4)°. In the rings *C*, the (CH_2_)_*n*_ moieties are all displaced to the same side of ring *B*, in the direction opposite to the sulfur atom (Fig. 4 ▸).

## 3. Supra­molecular features

None of the compounds contains a classical hydrogen-bond donor, and so the mol­ecular packing must be inter­preted in terms of other ‘weak’ inter­actions. The most obvious of these are ‘weak’ C—H⋯N hydrogen bonds, mostly involving the nitro­gen atom of the nitrile group; however, it is a moot point whether these represent significant inter­actions or simply the exposed nature of the one-coordinated nitro­gen atoms. Each compound displays two such contacts.

**Figure 5:**
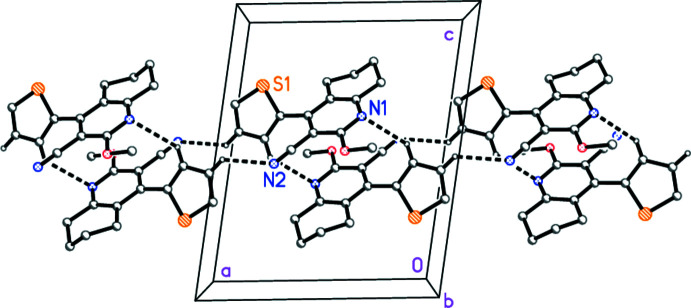
The mol­ecular packing of compound **1a**, viewed parallel to the *b* axis, showing the ‘weak’ hydrogen bonds (drawn as dashed bonds). Atom labels indicate the asymmetric unit.

For compound **1a**, the two hydrogen bonds (Table 4 ▸), one to each of the two nitro­gen atoms, combine to form a one-dimensional assembly parallel to the *a* axis (Fig. 5 ▸). Both operators involve inversion. Further contacts may be identified: a possible stacking of two rings *B*, as seen in the Figure [inter­centroid distance 3.6516 (6) Å, offset 1.23 Å, operator −*x* + 1, −*y* + 1, −*z* + 1]; a C—H⋯π contact from H6*B* to the centroid (*Cg*) of ring *A* (H⋯*Cg* = 2.90 Å, C—H⋯*Cg* = 143°, operator −*x* + 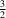 , *y* − 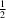 , −*z* + 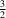 ); and a possible S⋯π contact (Ringer *et al.*, 2007 ▸; Daeffler *et al.*, 2012 ▸; Motherwell *et al.*, 2018 ▸) to ring *B* [S⋯centroid 3.5460 (5) Å, same operator −*x* + 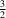 , *y* − 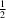 , −*z* + 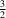 ], although this contact is markedly one-sided, with S1⋯C2 at 3.370 (1) Å shorter than the other contact distances.

Similarly, for compound **1c**, the two C—H⋯N hydrogen bonds, both *via* inversion operators but both involving the same acceptor N2 (Table 6 ▸, Fig. 7 ▸), lead to a one-dimensional structure parallel to [101]. However, whereas the H16⋯N2 inter­action is quite short, the contact from the methyl hydrogen atom H11*C* should probably be regarded as a borderline case.

**Figure 6:**
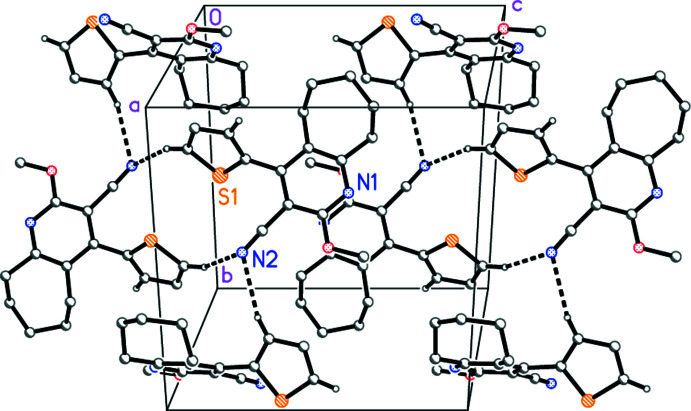
Part of the three-dimensional mol­ecular packing of compound **1b**, viewed perpendicular to (1 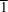 0), showing the ‘weak’ hydrogen bonds (drawn as dashed bonds). Atom labels indicate the asymmetric unit. Two inversion-symmetric substructures are shown, each with two further mol­ecules related by the 2_1_ axis.

**Figure 7:**
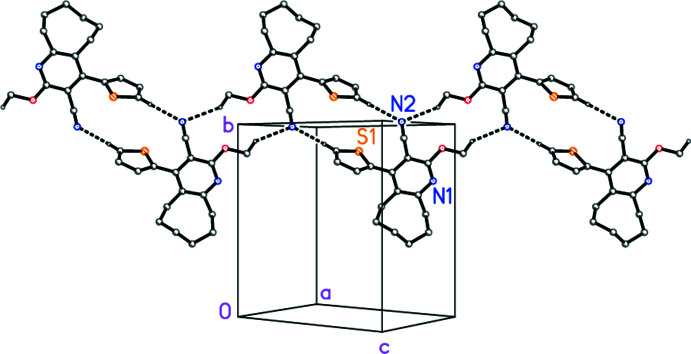
The mol­ecular packing of compound **1c**, viewed perpendicular to (10 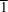 ), showing the ‘weak’ hydrogen bonds (drawn as dashed bonds). Atom labels indicate the asymmetric unit.

For compound **1b**, the two C—H⋯N hydrogen bonds again both involve N2 (Table 5 ▸), but the operators are different (one inversion centre and one 2_1_ screw axis). This leads to a complex three-dimensional structure, part of which is shown in Fig. 6 ▸. There is also a C—H⋯π contact from H7*B* to the centroid of ring *A* (H⋯*Cg* = 2.93 Å, C—H⋯*Cg* = 170°, operator *x* − 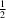 , −*y* + 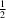 , *z* + 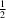 ).

## 4. Database survey

The searches employed the routine *ConQuest* (Bruno *et al.*, 2002 ▸), part of Version 2022.3.0 of the Cambridge Database (Groom *et al.*, 2016 ▸).

A search for the tetra­hydro­quinoline ring system corresponding to **1a** gave 69 hits (68 compounds excluding one repeat) with no substituents at the *sp* ^3^ carbon atoms. Almost all of these display a half-chair conformation of ring *C*; the only ordered example with a clear envelope conformation (five atoms approximately coplanar) was 2-amino-4-(1-methyl-1*H*-benzo[*d*]imidazol-2-yl)-5,6,7,8-tetra­hydro­quino­line-3-carbo­nitrile (refcode FIXGOL; Boulebd & Belfaitah, 2019 ▸).

A search for the cyclo­hepta­[*b*]pyridine subunit of **1b**, excluding ring systems with further annelation, led to 26 hits, corresponding (excluding repeats) to 23 compounds; eleven of these involve seven-membered rings with no further substit­uents. The hits include the natural products rupestine B (refcode SUGSAP; Su *et al.*, 2010 ▸) and D (refcode SUGSET; Su *et al.*, 2010 ▸, Zhang *et al.*, 2021 ▸). An analogous search for cyclo­octa­[*b*]pyridine derivatives (corresponding to **1c**) gave 19 hits for 18 unique compounds; in all cases, the eight-membered rings bear no further substituents. Both searches showed that the three or four central methyl­ene groups always lie on the same side of the plane of the pyridine-type ring (ring *B* in Section 2), as observed for **1b** and **1c** (Fig. 4 ▸). They also confirmed the general trend to wide bond angles in the (CH_2_)_*n*_ moieties.

## 5. Synthesis and crystallization

Compounds **1a**–**c** were prepared following our literature procedures (Elgemeie *et al.*, 1991 ▸) and crystallized from ethanol.

## 6. Refinement

Crystal data, data collection and structure refinement details are summarized in Table 7 ▸. Methyl groups were included as idealized rigid groups allowed to rotate but not tip (C—H = 0.98 Å, H—C—H = 109.5°). Other hydrogen atoms were included using a riding model starting from calculated positions (C—H_aromatic_ = 0.95 Å, C—H_methyl­ene_ = 0.98 Å, C—H_methine_ = 1.00 Å). The *U*_iso_(H) values were fixed at 1.5 × *U*_eq_ of the parent carbon atoms for methyls and 1.2 × *U*_eq_ for other hydrogens.

The structure of **1b** was refined as a two-component twin using the HKLF 5 method (Sheldrick, 2015*a* ▸). The crystal was non-merohedrally twinned by 180° rotation about the vector (**a** + **c**). The scale factor (BASF, the relative volume of the smaller component) refined to 0.4982 (8). The thienyl group is disordered by *ca* 180° rotation about the bond C4—C12. The occupation factor of the major disorder component refined to 0.917 (2).

In the structure of **1c**, the atoms C7 and C8 of the eight-membered ring are disordered over two positions; the relative occupation factors refined to 0.899 and 0.101 (3).

For both disordered structures, appropriate restraints (*e.g.* setting bond lengths and angles of the disorder components to be approximately equal, command SAME) were employed to improve stability of refinement, but the dimensions of disordered groups (especially the minor components) should be inter­preted with caution.

## Supplementary Material

Crystal structure: contains datablock(s) 1a, 1b, 1c, global. DOI: 10.1107/S2056989023001883/yz2030sup1.cif

Structure factors: contains datablock(s) 1a. DOI: 10.1107/S2056989023001883/yz20301asup2.hkl

Structure factors: contains datablock(s) 1b. DOI: 10.1107/S2056989023001883/yz20301bsup3.hkl

Structure factors: contains datablock(s) 1c. DOI: 10.1107/S2056989023001883/yz20301csup4.hkl

Supporting information file. DOI: 10.1107/S2056989023001883/yz20301asup5.cml

Supporting information file. DOI: 10.1107/S2056989023001883/yz20301bsup6.cml

Supporting information file. DOI: 10.1107/S2056989023001883/yz20301csup7.cml

CCDC references: 2245466, 2245465, 2245464

Additional supporting information:  crystallographic information; 3D view; checkCIF report

## Figures and Tables

**Figure 2:**
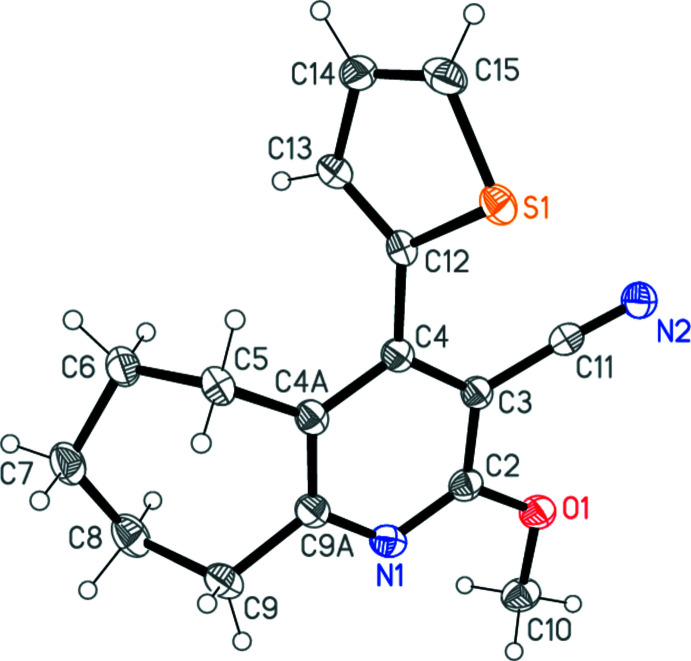
The mol­ecule of **1b** in the crystal. Ellipsoids represent 50% probability levels. The minor position [occupation factor 0.083 (3)] of the disordered thienyl group is omitted.

**Figure 3:**
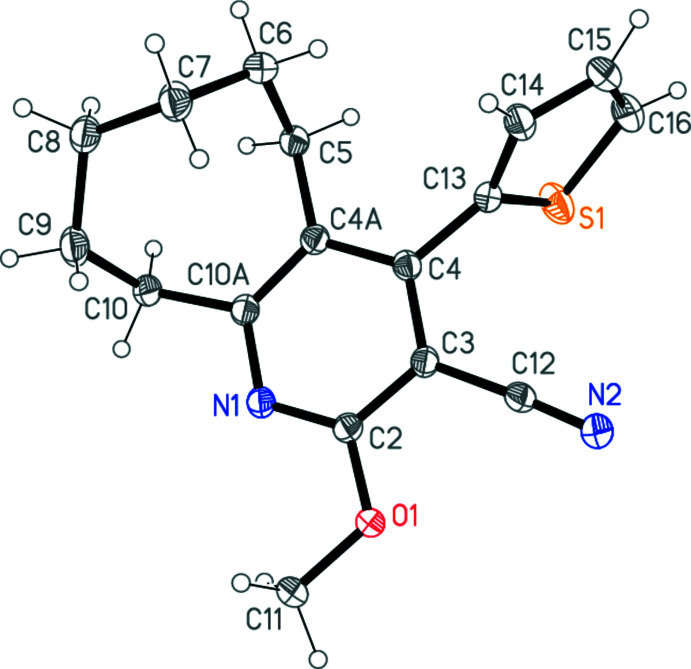
The mol­ecule of **1c** in the crystal. Ellipsoids represent 50% probability levels. The minor positions [occupation factor 0.101 (3)] of the disordered atoms C7 and C8 are omitted.

**Table 1:** Selected bond and torsion angles (°) for **1a** [Chem scheme1]

C7—C6—C5	110.08 (10)	N1—C8*A*—C8	113.91 (9)
C6—C7—C8	110.00 (9)		
			
C8*A*—C4*A*—C5—C6	18.12 (15)	C6—C7—C8—C8*A*	−42.62 (14)
C4*A*—C5—C6—C7	−50.80 (13)	C5—C4*A*—C8*A*—C8	2.50 (16)
C5—C6—C7—C8	63.65 (13)	C7—C8—C8*A*—C4*A*	10.21 (15)

**Table 2:** Selected bond and torsion angles (°) for **1b** [Chem scheme1]

C7—C6—C5	113.31 (10)	C7—C8—C9	115.55 (10)
C6—C7—C8	115.70 (10)	N1—C9*A*—C9	115.16 (10)
			
C9*A*—C4*A*—C5—C6	−68.49 (13)	C7—C8—C9—C9*A*	−78.94 (13)
C4*A*—C5—C6—C7	81.19 (12)	C5—C4*A*—C9*A*—C9	2.62 (15)
C5—C6—C7—C8	−60.92 (14)	C8—C9—C9*A*—C4*A*	62.64 (14)
C6—C7—C8—C9	61.40 (14)		

**Table 3:** Selected bond and torsion angles (°) for **1c** [Chem scheme1]

C7—C6—C5	115.95 (10)	C10—C9—C8	115.98 (11)
C6—C7—C8	114.70 (12)	N1—C10*A*—C10	114.11 (10)
C7—C8—C9	115.32 (12)		
			
C10*A*—C4*A*—C5—C6	91.62 (13)	C7—C8—C9—C10	−72.30 (16)
C4*A*—C5—C6—C7	−49.08 (15)	C8—C9—C10—C10*A*	78.19 (15)
C5—C6—C7—C8	−56.74 (16)	C5—C4*A*—C10*A*—C10	−0.92 (17)
C6—C7—C8—C9	100.76 (15)	C9—C10—C10*A*—C4*A*	−81.90 (15)

**Table 4:** Hydrogen-bond geometry (Å, °) for **1a** [Chem scheme1]

*D*—H⋯*A*	*D*—H	H⋯*A*	*D*⋯*A*	*D*—H⋯*A*
C12—H12⋯N1^i^	0.95	2.65	3.3499 (14)	131
C13—H13⋯N2^ii^	0.95	2.63	3.3503 (14)	133

Symmetry codes: (i) 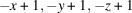 ; (ii) 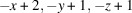 .

**Table 5:** Hydrogen-bond geometry (Å, °) for **1b** [Chem scheme1]

*D*—H⋯*A*	*D*—H	H⋯*A*	*D*⋯*A*	*D*—H⋯*A*
C13—H13⋯N2^i^	0.95	2.60	3.524 (3)	164
C15—H15⋯N2^ii^	0.95	2.53	3.3941 (19)	152

Symmetry codes: (i) 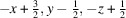 ; (ii) 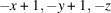 .

**Table 6:** Hydrogen-bond geometry (Å, °) for **1c** [Chem scheme1]

*D*—H⋯*A*	*D*—H	H⋯*A*	*D*⋯*A*	*D*—H⋯*A*
C11—H11*C*⋯N2^i^	0.98	2.69	3.4864 (16)	138
C16—H16⋯N2^ii^	0.95	2.41	3.3379 (17)	167

Symmetry codes: (i) 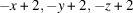 ; (ii) 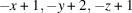 .

**Table 7:** Experimental details

	**1a**	**1b**	**1c**
Crystal data			
Chemical formula	C_15_H_14_N_2_OS	C_16_H_16_N_2_OS	C_17_H_18_N_2_OS
*M*_r_	270.34	284.37	298.39
Crystal system, space group	Monoclinic, *P*2_1_/*n*	Monoclinic, *P*2_1_/*n*	Monoclinic, *P*2_1_/*c*
Temperature (K)	100	100	100
*a*, *b*, *c* (Å)	10.85636 (13), 9.1857 (1), 13.31001 (16)	8.68561 (17), 13.7435 (2), 12.0379 (2)	9.87736 (14), 12.51312 (19), 12.53915 (19)
β (°)	98.7757 (12)	99.9254 (18)	102.9861 (14)
*V* (Å^3^)	1311.78 (3)	1415.47 (5)	1510.16 (4)
*Z*	4	4	4
Radiation type	Cu *K*α	Cu *K*α	Cu *K*α
μ (mm^−1^)	2.13	2.00	1.90
Crystal size (mm)	0.10 × 0.08 × 0.03	0.15 × 0.08 × 0.03	0.12 × 0.06 × 0.05

Data collection			
Diffractometer	XtaLAB Synergy	XtaLAB Synergy	XtaLAB Synergy
Absorption correction	Multi-scan (*CrysAlis PRO*; Rigaku OD, 2022 ▸)	Multi-scan (*CrysAlis PRO*; Rigaku OD, 2022 ▸)	Multi-scan (*CrysAlis PRO*; Rigaku OD, 2022 ▸)
*T*_min_, *T*_max_	0.820, 1.000	0.835, 1.000	0.840, 1.000
No. of measured, independent and observed [*I* > 2σ(*I*)] reflections	59253, 2774, 2663	5190, 5190, 4977	68720, 3210, 3087
*R*_int_	0.032	–	0.029
(sin θ/λ)_max_ (Å^−1^)	0.634	0.634	0.633

Refinement			
*R*[*F* ^2^ > 2σ(*F* ^2^)], *wR*(*F* ^2^), *S*	0.026, 0.071, 1.07	0.029, 0.077, 1.07	0.034, 0.091, 1.10
No. of reflections	2774	5190	3210
No. of parameters	173	204	200
No. of restraints	0	51	5
H-atom treatment	H-atom parameters constrained	H-atom parameters constrained	H-atom parameters constrained
Δρ_max_, Δρ_min_ (e Å^−3^)	0.28, −0.24	0.20, −0.33	0.28, −0.48

Computer programs: *CrysAlis PRO* (Rigaku OD, 2022 ▸), *SHELXT* (Sheldrick, 2015*a* ▸), *SHELXL2018/3* (Sheldrick, 2015*b* ▸) and *XP* (Siemens, 1994 ▸).

## supplementary crystallographic information

### 2-Methoxy-4-(thiophen-2-yl)-5,6,7,8-tetrahydroquinoline-3-carbonitrile (1a) Crystal data

**Table:**

C_15_H_14_N_2_OS	*F*(000) = 568
*M**_r_* = 270.34	*D*_x_ = 1.369 Mg m^−^^3^
Monoclinic, *P*2_1_/*n*	Cu *K*α radiation, λ = 1.54184 Å
*a* = 10.85636 (13) Å	Cell parameters from 35765 reflections
*b* = 9.1857 (1) Å	θ = 4.9–77.1°
*c* = 13.31001 (16) Å	µ = 2.13 mm^−^^1^
β = 98.7757 (12)°	*T* = 100 K
*V* = 1311.78 (3) Å^3^	Irregular, colourless
*Z* = 4	0.10 × 0.08 × 0.03 mm

### 2-Methoxy-4-(thiophen-2-yl)-5,6,7,8-tetrahydroquinoline-3-carbonitrile (1a) Data collection

**Table:**

XtaLAB Synergy diffractometer	2774 independent reflections
Radiation source: micro-focus sealed X-ray tube, PhotonJet (Cu) X-ray Source	2663 reflections with *I* > 2σ(*I*)
Mirror monochromator	*R*_int_ = 0.032
Detector resolution: 10.0000 pixels mm^-1^	θ_max_ = 77.7°, θ_min_ = 4.9°
ω scans	*h* = −13→13
Absorption correction: multi-scan (CrysAlisPro; Rigaku OD, 2022)	*k* = −11→11
*T*_min_ = 0.820, *T*_max_ = 1.000	*l* = −16→16
59253 measured reflections	

### 2-Methoxy-4-(thiophen-2-yl)-5,6,7,8-tetrahydroquinoline-3-carbonitrile (1a) Refinement

**Table:**

Refinement on *F*^2^	Primary atom site location: dual
Least-squares matrix: full	Hydrogen site location: inferred from neighbouring sites
*R*[*F*^2^ > 2σ(*F*^2^)] = 0.026	H-atom parameters constrained
*wR*(*F*^2^) = 0.071	*w* = 1/[σ^2^(*F*_o_^2^) + (0.0343*P*)^2^ + 0.5681*P*] where *P* = (*F*_o_^2^ + 2*F*_c_^2^)/3
*S* = 1.07	(Δ/σ)_max_ < 0.001
2774 reflections	Δρ_max_ = 0.28 e Å^−^^3^
173 parameters	Δρ_min_ = −0.24 e Å^−^^3^
0 restraints	

### 2-Methoxy-4-(thiophen-2-yl)-5,6,7,8-tetrahydroquinoline-3-carbonitrile (1a) Special details

**Table:**

Geometry. All esds (except the esd in the dihedral angle between two l.s. planes) are estimated using the full covariance matrix. The cell esds are taken into account individually in the estimation of esds in distances, angles and torsion angles; correlations between esds in cell parameters are only used when they are defined by crystal symmetry. An approximate (isotropic) treatment of cell esds is used for estimating esds involving l.s. planes.

### 2-Methoxy-4-(thiophen-2-yl)-5,6,7,8-tetrahydroquinoline-3-carbonitrile (1a) Fractional atomic coordinates and isotropic or equivalent isotropic displacement parameters (Å^2^)

**Table:**

	*x*	*y*	*z*	*U*_iso_*/*U*_eq_	
N1	0.41901 (8)	0.65182 (10)	0.62167 (7)	0.01531 (18)	
C2	0.49881 (9)	0.69755 (11)	0.56350 (8)	0.0145 (2)	
C3	0.61817 (9)	0.63623 (11)	0.56496 (8)	0.0145 (2)	
C4	0.65292 (9)	0.51730 (11)	0.62923 (8)	0.0143 (2)	
C4A	0.56608 (10)	0.46444 (11)	0.68941 (8)	0.0156 (2)	
C5	0.59305 (10)	0.33045 (13)	0.75609 (8)	0.0201 (2)	
H5A	0.649271	0.357815	0.818958	0.024*	
H5B	0.636738	0.257414	0.719674	0.024*	
C6	0.47503 (11)	0.26226 (13)	0.78460 (9)	0.0235 (2)	
H6A	0.425966	0.217090	0.723760	0.028*	
H6B	0.497721	0.185108	0.836031	0.028*	
C7	0.39682 (12)	0.37797 (14)	0.82721 (9)	0.0261 (3)	
H7A	0.446984	0.425907	0.886394	0.031*	
H7B	0.323728	0.331799	0.850455	0.031*	
C8	0.35304 (10)	0.49113 (13)	0.74576 (9)	0.0204 (2)	
H8A	0.281165	0.451055	0.699260	0.024*	
H8B	0.323737	0.578340	0.778891	0.024*	
C8A	0.45209 (10)	0.53687 (12)	0.68392 (8)	0.0156 (2)	
C9	0.35965 (10)	0.89244 (12)	0.50857 (9)	0.0206 (2)	
H9A	0.350748	0.972299	0.459164	0.031*	
H9B	0.365752	0.932339	0.577434	0.031*	
H9C	0.286883	0.828279	0.495114	0.031*	
O1	0.47092 (7)	0.81060 (8)	0.49971 (6)	0.01807 (17)	
C10	0.70092 (10)	0.70570 (11)	0.50540 (8)	0.0164 (2)	
N2	0.76402 (9)	0.76686 (11)	0.45769 (8)	0.0230 (2)	
S1	0.87568 (2)	0.41082 (3)	0.74141 (2)	0.02107 (9)	
C11	0.77753 (9)	0.45283 (11)	0.63009 (8)	0.0152 (2)	
C12	0.83478 (10)	0.42163 (11)	0.54747 (8)	0.0171 (2)	
H12	0.796800	0.437646	0.479223	0.021*	
C13	0.95620 (10)	0.36312 (12)	0.57419 (9)	0.0199 (2)	
H13	1.007563	0.334334	0.525951	0.024*	
C14	0.99099 (10)	0.35277 (12)	0.67641 (9)	0.0218 (2)	
H14	1.069755	0.317592	0.707976	0.026*	

### 2-Methoxy-4-(thiophen-2-yl)-5,6,7,8-tetrahydroquinoline-3-carbonitrile (1a) Atomic displacement parameters (Å^2^)

**Table:**

	*U*^11^	*U*^22^	*U*^33^	*U*^12^	*U*^13^	*U*^23^
N1	0.0144 (4)	0.0156 (4)	0.0161 (4)	−0.0005 (3)	0.0031 (3)	−0.0020 (3)
C2	0.0155 (5)	0.0132 (5)	0.0144 (5)	−0.0002 (4)	0.0015 (4)	−0.0016 (4)
C3	0.0145 (5)	0.0147 (5)	0.0145 (5)	−0.0006 (4)	0.0030 (4)	−0.0014 (4)
C4	0.0144 (5)	0.0147 (5)	0.0137 (5)	0.0000 (4)	0.0019 (4)	−0.0022 (4)
C4A	0.0172 (5)	0.0159 (5)	0.0140 (5)	−0.0005 (4)	0.0034 (4)	−0.0001 (4)
C5	0.0201 (5)	0.0209 (5)	0.0203 (5)	0.0022 (4)	0.0062 (4)	0.0059 (4)
C6	0.0243 (6)	0.0227 (6)	0.0256 (6)	0.0013 (5)	0.0103 (5)	0.0083 (5)
C7	0.0277 (6)	0.0296 (6)	0.0242 (6)	0.0029 (5)	0.0140 (5)	0.0065 (5)
C8	0.0191 (5)	0.0221 (5)	0.0220 (5)	0.0005 (4)	0.0095 (4)	0.0011 (4)
C8A	0.0164 (5)	0.0166 (5)	0.0141 (5)	−0.0017 (4)	0.0038 (4)	−0.0021 (4)
C9	0.0167 (5)	0.0167 (5)	0.0291 (6)	0.0046 (4)	0.0059 (4)	0.0036 (4)
O1	0.0164 (4)	0.0166 (4)	0.0221 (4)	0.0040 (3)	0.0055 (3)	0.0046 (3)
C10	0.0159 (5)	0.0145 (5)	0.0187 (5)	0.0040 (4)	0.0025 (4)	0.0007 (4)
N2	0.0202 (5)	0.0218 (5)	0.0288 (5)	0.0039 (4)	0.0097 (4)	0.0070 (4)
S1	0.01736 (14)	0.02812 (16)	0.01744 (14)	0.00374 (10)	0.00170 (10)	0.00578 (10)
C11	0.0148 (5)	0.0141 (5)	0.0167 (5)	0.0000 (4)	0.0020 (4)	0.0019 (4)
C12	0.0164 (5)	0.0165 (5)	0.0188 (5)	0.0000 (4)	0.0036 (4)	0.0007 (4)
C13	0.0168 (5)	0.0176 (5)	0.0263 (6)	0.0019 (4)	0.0069 (4)	0.0016 (4)
C14	0.0152 (5)	0.0212 (5)	0.0292 (6)	0.0029 (4)	0.0041 (4)	0.0072 (4)

### 2-Methoxy-4-(thiophen-2-yl)-5,6,7,8-tetrahydroquinoline-3-carbonitrile (1a) Geometric parameters (Å, º)

**Table:**

N1—C2	1.3156 (14)	C7—H7B	0.9900
N1—C8A	1.3563 (14)	C8—C8A	1.5102 (14)
C2—O1	1.3463 (13)	C8—H8A	0.9900
C2—C3	1.4104 (14)	C8—H8B	0.9900
C3—C4	1.4032 (14)	C9—O1	1.4427 (12)
C3—C10	1.4353 (14)	C9—H9A	0.9800
C4—C4A	1.4130 (14)	C9—H9B	0.9800
C4—C11	1.4752 (14)	C9—H9C	0.9800
C4A—C8A	1.3971 (15)	C10—N2	1.1492 (15)
C4A—C5	1.5195 (14)	S1—C14	1.7120 (12)
C5—C6	1.5252 (15)	S1—C11	1.7314 (11)
C5—H5A	0.9900	C11—C12	1.3734 (15)
C5—H5B	0.9900	C12—C13	1.4177 (15)
C6—C7	1.5229 (16)	C12—H12	0.9500
C6—H6A	0.9900	C13—C14	1.3586 (17)
C6—H6B	0.9900	C13—H13	0.9500
C7—C8	1.5246 (16)	C14—H14	0.9500
C7—H7A	0.9900		
			
C2—N1—C8A	118.12 (9)	C8A—C8—C7	113.99 (9)
N1—C2—O1	120.82 (9)	C8A—C8—H8A	108.8
N1—C2—C3	123.40 (10)	C7—C8—H8A	108.8
O1—C2—C3	115.75 (9)	C8A—C8—H8B	108.8
C4—C3—C2	118.70 (9)	C7—C8—H8B	108.8
C4—C3—C10	123.38 (9)	H8A—C8—H8B	107.6
C2—C3—C10	117.78 (9)	N1—C8A—C4A	123.55 (9)
C3—C4—C4A	118.20 (9)	N1—C8A—C8	113.91 (9)
C3—C4—C11	118.57 (9)	C4A—C8A—C8	122.53 (10)
C4A—C4—C11	123.23 (9)	O1—C9—H9A	109.5
C8A—C4A—C4	117.96 (9)	O1—C9—H9B	109.5
C8A—C4A—C5	120.43 (9)	H9A—C9—H9B	109.5
C4—C4A—C5	121.58 (9)	O1—C9—H9C	109.5
C4A—C5—C6	112.56 (9)	H9A—C9—H9C	109.5
C4A—C5—H5A	109.1	H9B—C9—H9C	109.5
C6—C5—H5A	109.1	C2—O1—C9	117.45 (8)
C4A—C5—H5B	109.1	N2—C10—C3	176.95 (11)
C6—C5—H5B	109.1	C14—S1—C11	92.23 (5)
H5A—C5—H5B	107.8	C12—C11—C4	127.16 (10)
C7—C6—C5	110.08 (10)	C12—C11—S1	110.13 (8)
C7—C6—H6A	109.6	C4—C11—S1	122.68 (8)
C5—C6—H6A	109.6	C11—C12—C13	113.28 (10)
C7—C6—H6B	109.6	C11—C12—H12	123.4
C5—C6—H6B	109.6	C13—C12—H12	123.4
H6A—C6—H6B	108.2	C14—C13—C12	112.56 (10)
C6—C7—C8	110.00 (9)	C14—C13—H13	123.7
C6—C7—H7A	109.7	C12—C13—H13	123.7
C8—C7—H7A	109.7	C13—C14—S1	111.79 (8)
C6—C7—H7B	109.7	C13—C14—H14	124.1
C8—C7—H7B	109.7	S1—C14—H14	124.1
H7A—C7—H7B	108.2		
			
C8A—N1—C2—O1	179.60 (9)	C2—N1—C8A—C8	−178.79 (9)
C8A—N1—C2—C3	−2.09 (15)	C4—C4A—C8A—N1	2.20 (16)
N1—C2—C3—C4	2.08 (16)	C5—C4A—C8A—N1	−176.07 (10)
O1—C2—C3—C4	−179.53 (9)	C4—C4A—C8A—C8	−179.22 (9)
N1—C2—C3—C10	−173.74 (9)	C5—C4A—C8A—C8	2.50 (16)
O1—C2—C3—C10	4.65 (14)	C7—C8—C8A—N1	−171.09 (10)
C2—C3—C4—C4A	0.13 (15)	C7—C8—C8A—C4A	10.21 (15)
C10—C3—C4—C4A	175.70 (9)	N1—C2—O1—C9	9.04 (14)
C2—C3—C4—C11	179.62 (9)	C3—C2—O1—C9	−169.39 (9)
C10—C3—C4—C11	−4.81 (15)	C3—C4—C11—C12	−44.17 (16)
C3—C4—C4A—C8A	−2.10 (15)	C4A—C4—C11—C12	135.29 (12)
C11—C4—C4A—C8A	178.44 (9)	C3—C4—C11—S1	133.63 (9)
C3—C4—C4A—C5	176.16 (10)	C4A—C4—C11—S1	−46.91 (14)
C11—C4—C4A—C5	−3.30 (16)	C14—S1—C11—C12	0.06 (9)
C8A—C4A—C5—C6	18.12 (15)	C14—S1—C11—C4	−178.08 (9)
C4—C4A—C5—C6	−160.09 (10)	C4—C11—C12—C13	178.59 (10)
C4A—C5—C6—C7	−50.80 (13)	S1—C11—C12—C13	0.56 (12)
C5—C6—C7—C8	63.65 (13)	C11—C12—C13—C14	−1.09 (14)
C6—C7—C8—C8A	−42.62 (14)	C12—C13—C14—S1	1.11 (13)
C2—N1—C8A—C4A	−0.11 (15)	C11—S1—C14—C13	−0.68 (9)

### 2-Methoxy-4-(thiophen-2-yl)-5,6,7,8-tetrahydroquinoline-3-carbonitrile (1a) Hydrogen-bond geometry (Å, º)

**Table:**

*D*—H···*A*	*D*—H	H···*A*	*D*···*A*	*D*—H···*A*
C12—H12···N1^i^	0.95	2.65	3.3499 (14)	131
C13—H13···N2^ii^	0.95	2.63	3.3503 (14)	133

Symmetry codes: (i) −*x*+1, −*y*+1, −*z*+1; (ii) −*x*+2, −*y*+1, −*z*+1.

### 2-Methoxy-4-(thiophen-2-yl)-6,7,8,9-tetrahydro-5H-cyclohepta[b]pyridine-3-carbonitrile (1b) Crystal data

**Table:**

C_16_H_16_N_2_OS	*F*(000) = 600
*M**_r_* = 284.37	*D*_x_ = 1.334 Mg m^−^^3^
Monoclinic, *P*2_1_/*n*	Cu *K*α radiation, λ = 1.54184 Å
*a* = 8.68561 (17) Å	Cell parameters from 45134 reflections
*b* = 13.7435 (2) Å	θ = 4.9–76.7°
*c* = 12.0379 (2) Å	µ = 2.00 mm^−^^1^
β = 99.9254 (18)°	*T* = 100 K
*V* = 1415.47 (5) Å^3^	Plate, colourless
*Z* = 4	0.15 × 0.08 × 0.03 mm

### 2-Methoxy-4-(thiophen-2-yl)-6,7,8,9-tetrahydro-5H-cyclohepta[b]pyridine-3-carbonitrile (1b) Data collection

**Table:**

XtaLAB Synergy diffractometer	5190 measured reflections
Radiation source: micro-focus sealed X-ray tube, PhotonJet (Cu) X-ray Source	5190 independent reflections
Mirror monochromator	4977 reflections with *I* > 2σ(*I*)
Detector resolution: 10.0000 pixels mm^-1^	θ_max_ = 77.9°, θ_min_ = 4.9°
ω scans	*h* = −10→10
Absorption correction: multi-scan (CrysAlisPro; Rigaku OD, 2022)	*k* = −17→17
*T*_min_ = 0.835, *T*_max_ = 1.000	*l* = −15→15

### 2-Methoxy-4-(thiophen-2-yl)-6,7,8,9-tetrahydro-5H-cyclohepta[b]pyridine-3-carbonitrile (1b) Refinement

**Table:**

Refinement on *F*^2^	Primary atom site location: dual
Least-squares matrix: full	Hydrogen site location: inferred from neighbouring sites
*R*[*F*^2^ > 2σ(*F*^2^)] = 0.029	H-atom parameters constrained
*wR*(*F*^2^) = 0.077	*w* = 1/[σ^2^(*F*_o_^2^) + (0.0429*P*)^2^ + 0.218*P*] where *P* = (*F*_o_^2^ + 2*F*_c_^2^)/3
*S* = 1.07	(Δ/σ)_max_ = 0.001
5190 reflections	Δρ_max_ = 0.20 e Å^−^^3^
204 parameters	Δρ_min_ = −0.33 e Å^−^^3^
51 restraints	

### 2-Methoxy-4-(thiophen-2-yl)-6,7,8,9-tetrahydro-5H-cyclohepta[b]pyridine-3-carbonitrile (1b) Special details

**Table:**

Geometry. All esds (except the esd in the dihedral angle between two l.s. planes) are estimated using the full covariance matrix. The cell esds are taken into account individually in the estimation of esds in distances, angles and torsion angles; correlations between esds in cell parameters are only used when they are defined by crystal symmetry. An approximate (isotropic) treatment of cell esds is used for estimating esds involving l.s. planes.

### 2-Methoxy-4-(thiophen-2-yl)-6,7,8,9-tetrahydro-5H-cyclohepta[b]pyridine-3-carbonitrile (1b) Fractional atomic coordinates and isotropic or equivalent isotropic displacement parameters (Å^2^)

**Table:**

	*x*	*y*	*z*	*U*_iso_*/*U*_eq_	Occ. (<1)
N1	0.62391 (11)	0.40093 (7)	0.55582 (8)	0.0225 (2)	
C2	0.68755 (12)	0.44841 (8)	0.47970 (9)	0.0208 (2)	
C3	0.64622 (12)	0.43257 (7)	0.36319 (9)	0.0194 (2)	
C4A	0.46175 (12)	0.31252 (8)	0.40494 (9)	0.0202 (2)	
C5	0.33189 (13)	0.23967 (8)	0.37129 (9)	0.0234 (2)	
H5A	0.299458	0.241721	0.288350	0.028*	
H5B	0.240830	0.259286	0.405503	0.028*	
C6	0.37634 (15)	0.13462 (9)	0.40673 (11)	0.0283 (3)	
H6A	0.306161	0.089483	0.357744	0.034*	
H6B	0.484451	0.121942	0.394673	0.034*	
C7	0.36601 (15)	0.11332 (9)	0.52956 (11)	0.0292 (3)	
H7A	0.391137	0.043785	0.544622	0.035*	
H7B	0.256606	0.123530	0.540072	0.035*	
C8	0.47243 (15)	0.17420 (9)	0.61698 (11)	0.0307 (3)	
H8A	0.582141	0.161925	0.608561	0.037*	
H8B	0.460131	0.151458	0.692992	0.037*	
C9	0.44330 (14)	0.28469 (9)	0.61047 (9)	0.0265 (2)	
H9A	0.329164	0.296497	0.597463	0.032*	
H9B	0.488083	0.314069	0.684049	0.032*	
C9A	0.51199 (12)	0.33508 (8)	0.51922 (9)	0.0215 (2)	
O1	0.79988 (9)	0.51505 (6)	0.51257 (6)	0.02512 (18)	
C10	0.85462 (14)	0.52347 (9)	0.63198 (9)	0.0274 (2)	
H10A	0.939489	0.571270	0.645841	0.041*	
H10B	0.893140	0.460149	0.662292	0.041*	
H10C	0.768497	0.544735	0.669146	0.041*	
C11	0.72102 (12)	0.49002 (8)	0.28838 (9)	0.0206 (2)	
N2	0.78244 (11)	0.53879 (7)	0.23194 (8)	0.0256 (2)	
C4	0.53379 (12)	0.36142 (8)	0.32516 (9)	0.0191 (2)	
C12	0.4956 (4)	0.3372 (2)	0.20351 (15)	0.0192 (6)	0.9165 (18)
S1	0.41398 (4)	0.42140 (3)	0.10427 (4)	0.02356 (11)	0.9165 (18)
C13	0.5122 (4)	0.2478 (2)	0.1561 (3)	0.0241 (6)	0.9165 (18)
H13	0.554088	0.192012	0.197180	0.029*	0.9165 (18)
C14	0.45807 (17)	0.24944 (12)	0.03647 (12)	0.0265 (3)	0.9165 (18)
H14	0.461758	0.194492	−0.010884	0.032*	0.9165 (18)
C15	0.40077 (16)	0.33795 (15)	−0.00242 (11)	0.0276 (3)	0.9165 (18)
H15	0.358864	0.351468	−0.079069	0.033*	0.9165 (18)
S1'	0.4973 (14)	0.2438 (9)	0.1354 (9)	0.034 (3)*	0.0835 (18)
C12'	0.482 (5)	0.349 (2)	0.2035 (17)	0.021 (8)*	0.0835 (18)
C13'	0.416 (4)	0.413 (2)	0.128 (2)	0.079 (13)*	0.0835 (18)
H13'	0.391857	0.477403	0.148342	0.095*	0.0835 (18)
C14'	0.383 (2)	0.3797 (15)	0.0179 (16)	0.037 (5)*	0.0835 (18)
H14'	0.339750	0.418697	−0.044909	0.044*	0.0835 (18)
C15'	0.421 (2)	0.2852 (15)	0.0112 (14)	0.026 (4)*	0.0835 (18)
H15'	0.404855	0.247901	−0.056219	0.031*	0.0835 (18)

### 2-Methoxy-4-(thiophen-2-yl)-6,7,8,9-tetrahydro-5H-cyclohepta[b]pyridine-3-carbonitrile (1b) Atomic displacement parameters (Å^2^)

**Table:**

	*U*^11^	*U*^22^	*U*^33^	*U*^12^	*U*^13^	*U*^23^
N1	0.0234 (4)	0.0247 (4)	0.0197 (4)	0.0042 (4)	0.0047 (3)	0.0008 (4)
C2	0.0206 (5)	0.0195 (5)	0.0224 (5)	0.0034 (4)	0.0037 (4)	−0.0009 (4)
C3	0.0196 (5)	0.0183 (5)	0.0208 (5)	0.0025 (4)	0.0049 (4)	0.0014 (4)
C4A	0.0199 (5)	0.0194 (5)	0.0219 (5)	0.0035 (4)	0.0054 (4)	0.0034 (4)
C5	0.0236 (5)	0.0246 (5)	0.0226 (5)	−0.0018 (4)	0.0063 (4)	0.0042 (4)
C6	0.0339 (6)	0.0224 (5)	0.0315 (6)	−0.0015 (5)	0.0142 (5)	0.0026 (5)
C7	0.0338 (6)	0.0237 (5)	0.0326 (6)	0.0041 (5)	0.0131 (5)	0.0094 (5)
C8	0.0322 (6)	0.0322 (7)	0.0288 (6)	0.0051 (5)	0.0082 (5)	0.0127 (5)
C9	0.0292 (6)	0.0308 (6)	0.0211 (5)	0.0019 (5)	0.0085 (4)	0.0037 (4)
C9A	0.0221 (5)	0.0211 (5)	0.0222 (5)	0.0051 (4)	0.0065 (4)	0.0028 (4)
O1	0.0263 (4)	0.0268 (4)	0.0215 (4)	−0.0032 (3)	0.0018 (3)	−0.0030 (3)
C10	0.0296 (6)	0.0291 (6)	0.0214 (5)	0.0020 (5)	−0.0016 (4)	−0.0036 (4)
C11	0.0204 (5)	0.0199 (5)	0.0213 (5)	0.0005 (4)	0.0028 (4)	−0.0031 (4)
N2	0.0268 (5)	0.0255 (5)	0.0252 (5)	−0.0049 (4)	0.0062 (4)	−0.0012 (4)
C4	0.0192 (5)	0.0184 (5)	0.0199 (5)	0.0036 (4)	0.0041 (4)	0.0020 (4)
C12	0.0189 (9)	0.0194 (9)	0.0198 (9)	−0.0013 (7)	0.0047 (5)	0.0031 (5)
S1	0.02551 (18)	0.02386 (17)	0.02084 (18)	0.00268 (11)	0.00265 (12)	0.00618 (12)
C13	0.0299 (11)	0.0250 (10)	0.0180 (12)	−0.0021 (6)	0.0058 (9)	0.0021 (8)
C14	0.0302 (7)	0.0276 (7)	0.0230 (7)	−0.0086 (6)	0.0082 (5)	−0.0050 (6)
C15	0.0270 (6)	0.0379 (10)	0.0176 (6)	−0.0052 (6)	0.0032 (5)	0.0014 (6)

### 2-Methoxy-4-(thiophen-2-yl)-6,7,8,9-tetrahydro-5H-cyclohepta[b]pyridine-3-carbonitrile (1b) Geometric parameters (Å, º)

**Table:**

N1—C2	1.3212 (14)	O1—C10	1.4387 (13)
N1—C9A	1.3455 (15)	C10—H10A	0.9800
C2—O1	1.3470 (13)	C10—H10B	0.9800
C2—C3	1.4035 (15)	C10—H10C	0.9800
C3—C4	1.4019 (15)	C11—N2	1.1490 (14)
C3—C11	1.4348 (14)	C4—C12'	1.466 (19)
C4A—C9A	1.4043 (15)	C4—C12	1.482 (2)
C4A—C4	1.4045 (14)	C12—C13	1.373 (4)
C4A—C5	1.5104 (15)	C12—S1	1.725 (3)
C5—C6	1.5358 (16)	S1—C15	1.7109 (18)
C5—H5A	0.9900	C13—C14	1.436 (3)
C5—H5B	0.9900	C13—H13	0.9500
C6—C7	1.5251 (16)	C14—C15	1.366 (2)
C6—H6A	0.9900	C14—H14	0.9500
C6—H6B	0.9900	C15—H15	0.9500
C7—C8	1.5253 (19)	S1'—C15'	1.630 (17)
C7—H7A	0.9900	S1'—C12'	1.67 (2)
C7—H7B	0.9900	C12'—C13'	1.33 (2)
C8—C9	1.5393 (17)	C13'—C14'	1.39 (2)
C8—H8A	0.9900	C13'—H13'	0.9500
C8—H8B	0.9900	C14'—C15'	1.345 (19)
C9—C9A	1.5060 (14)	C14'—H14'	0.9500
C9—H9A	0.9900	C15'—H15'	0.9500
C9—H9B	0.9900		
			
C2—N1—C9A	118.04 (10)	N1—C9A—C9	115.16 (10)
N1—C2—O1	120.04 (10)	C4A—C9A—C9	121.18 (10)
N1—C2—C3	123.43 (10)	C2—O1—C10	116.49 (9)
O1—C2—C3	116.52 (9)	O1—C10—H10A	109.5
C4—C3—C2	118.58 (10)	O1—C10—H10B	109.5
C4—C3—C11	122.97 (10)	H10A—C10—H10B	109.5
C2—C3—C11	118.46 (10)	O1—C10—H10C	109.5
C9A—C4A—C4	117.67 (10)	H10A—C10—H10C	109.5
C9A—C4A—C5	120.04 (9)	H10B—C10—H10C	109.5
C4—C4A—C5	122.27 (10)	N2—C11—C3	177.19 (12)
C4A—C5—C6	114.10 (9)	C3—C4—C4A	118.54 (10)
C4A—C5—H5A	108.7	C3—C4—C12'	119.0 (16)
C6—C5—H5A	108.7	C4A—C4—C12'	122.1 (16)
C4A—C5—H5B	108.7	C3—C4—C12	120.25 (15)
C6—C5—H5B	108.7	C4A—C4—C12	121.19 (15)
H5A—C5—H5B	107.6	C13—C12—C4	126.1 (2)
C7—C6—C5	113.31 (10)	C13—C12—S1	111.88 (18)
C7—C6—H6A	108.9	C4—C12—S1	121.9 (2)
C5—C6—H6A	108.9	C15—S1—C12	92.11 (10)
C7—C6—H6B	108.9	C12—C13—C14	111.3 (2)
C5—C6—H6B	108.9	C12—C13—H13	124.4
H6A—C6—H6B	107.7	C14—C13—H13	124.4
C6—C7—C8	115.70 (10)	C15—C14—C13	113.24 (17)
C6—C7—H7A	108.4	C15—C14—H14	123.4
C8—C7—H7A	108.4	C13—C14—H14	123.4
C6—C7—H7B	108.4	C14—C15—S1	111.49 (10)
C8—C7—H7B	108.4	C14—C15—H15	124.3
H7A—C7—H7B	107.4	S1—C15—H15	124.3
C7—C8—C9	115.55 (10)	C15'—S1'—C12'	95.4 (12)
C7—C8—H8A	108.4	C13'—C12'—C4	129 (2)
C9—C8—H8A	108.4	C13'—C12'—S1'	107.5 (16)
C7—C8—H8B	108.4	C4—C12'—S1'	123.8 (18)
C9—C8—H8B	108.4	C12'—C13'—C14'	115 (2)
H8A—C8—H8B	107.5	C12'—C13'—H13'	122.3
C9A—C9—C8	114.11 (9)	C14'—C13'—H13'	122.3
C9A—C9—H9A	108.7	C15'—C14'—C13'	111.4 (18)
C8—C9—H9A	108.7	C15'—C14'—H14'	124.3
C9A—C9—H9B	108.7	C13'—C14'—H14'	124.3
C8—C9—H9B	108.7	C14'—C15'—S1'	110.3 (13)
H9A—C9—H9B	107.6	C14'—C15'—H15'	124.9
N1—C9A—C4A	123.66 (10)	S1'—C15'—H15'	124.9
			
C9A—N1—C2—O1	179.93 (9)	C9A—C4A—C4—C3	−2.71 (14)
C9A—N1—C2—C3	−1.04 (16)	C5—C4A—C4—C3	175.84 (9)
N1—C2—C3—C4	−1.29 (16)	C9A—C4A—C4—C12'	−175.4 (14)
O1—C2—C3—C4	177.77 (9)	C5—C4A—C4—C12'	3.2 (14)
N1—C2—C3—C11	178.70 (10)	C9A—C4A—C4—C12	175.80 (17)
O1—C2—C3—C11	−2.24 (14)	C5—C4A—C4—C12	−5.6 (2)
C9A—C4A—C5—C6	−68.49 (13)	C3—C4—C12—C13	119.7 (3)
C4—C4A—C5—C6	112.99 (11)	C4A—C4—C12—C13	−58.8 (4)
C4A—C5—C6—C7	81.19 (12)	C3—C4—C12—S1	−63.4 (3)
C5—C6—C7—C8	−60.92 (14)	C4A—C4—C12—S1	118.13 (19)
C6—C7—C8—C9	61.40 (14)	C13—C12—S1—C15	0.0 (2)
C7—C8—C9—C9A	−78.94 (13)	C4—C12—S1—C15	−177.3 (2)
C2—N1—C9A—C4A	1.50 (16)	C4—C12—C13—C14	177.7 (3)
C2—N1—C9A—C9	−179.26 (9)	S1—C12—C13—C14	0.5 (3)
C4—C4A—C9A—N1	0.40 (15)	C12—C13—C14—C15	−1.0 (3)
C5—C4A—C9A—N1	−178.19 (10)	C13—C14—C15—S1	1.0 (2)
C4—C4A—C9A—C9	−178.79 (10)	C12—S1—C15—C14	−0.58 (14)
C5—C4A—C9A—C9	2.62 (15)	C3—C4—C12'—C13'	−59 (3)
C8—C9—C9A—N1	−116.61 (11)	C4A—C4—C12'—C13'	113 (2)
C8—C9—C9A—C4A	62.64 (14)	C3—C4—C12'—S1'	122 (2)
N1—C2—O1—C10	5.86 (14)	C4A—C4—C12'—S1'	−66 (3)
C3—C2—O1—C10	−173.24 (9)	C15'—S1'—C12'—C13'	1.0 (16)
C2—C3—C4—C4A	3.15 (15)	C15'—S1'—C12'—C4	−180 (3)
C11—C3—C4—C4A	−176.84 (9)	C4—C12'—C13'—C14'	179 (4)
C2—C3—C4—C12'	176.0 (15)	S1'—C12'—C13'—C14'	−2 (2)
C11—C3—C4—C12'	−4.0 (15)	C12'—C13'—C14'—C15'	3 (3)
C2—C3—C4—C12	−175.38 (17)	C13'—C14'—C15'—S1'	−2 (3)
C11—C3—C4—C12	4.6 (2)	C12'—S1'—C15'—C14'	1 (2)

### 2-Methoxy-4-(thiophen-2-yl)-6,7,8,9-tetrahydro-5H-cyclohepta[b]pyridine-3-carbonitrile (1b) Hydrogen-bond geometry (Å, º)

**Table:**

*D*—H···*A*	*D*—H	H···*A*	*D*···*A*	*D*—H···*A*
C13—H13···N2^i^	0.95	2.60	3.524 (3)	164
C15—H15···N2^ii^	0.95	2.53	3.3941 (19)	152

Symmetry codes: (i) −*x*+3/2, *y*−1/2, −*z*+1/2; (ii) −*x*+1, −*y*+1, −*z*.

### 2-Methoxy-4-(thiophen-2-yl)-5,6,7,8,9,10-hexahydro-cycloocta[b]pyridine-3-carbonitrile (1c) Crystal data

**Table:**

C_17_H_18_N_2_OS	*F*(000) = 632
*M**_r_* = 298.39	*D*_x_ = 1.312 Mg m^−^^3^
Monoclinic, *P*2_1_/*c*	Cu *K*α radiation, λ = 1.54184 Å
*a* = 9.87736 (14) Å	Cell parameters from 43890 reflections
*b* = 12.51312 (19) Å	θ = 4.6–77.4°
*c* = 12.53915 (19) Å	µ = 1.90 mm^−^^1^
β = 102.9861 (14)°	*T* = 100 K
*V* = 1510.16 (4) Å^3^	Block, colourless
*Z* = 4	0.12 × 0.06 × 0.05 mm

### 2-Methoxy-4-(thiophen-2-yl)-5,6,7,8,9,10-hexahydro-cycloocta[b]pyridine-3-carbonitrile (1c) Data collection

**Table:**

XtaLAB Synergy diffractometer	3210 independent reflections
Radiation source: micro-focus sealed X-ray tube, PhotonJet (Cu) X-ray Source	3087 reflections with *I* > 2σ(*I*)
Mirror monochromator	*R*_int_ = 0.029
Detector resolution: 10.0000 pixels mm^-1^	θ_max_ = 77.6°, θ_min_ = 4.6°
ω scans	*h* = −12→12
Absorption correction: multi-scan (CrysAlisPro; Rigaku OD, 2022)	*k* = −15→15
*T*_min_ = 0.840, *T*_max_ = 1.000	*l* = −15→15
68720 measured reflections	

### 2-Methoxy-4-(thiophen-2-yl)-5,6,7,8,9,10-hexahydro-cycloocta[b]pyridine-3-carbonitrile (1c) Refinement

**Table:**

Refinement on *F*^2^	Primary atom site location: dual
Least-squares matrix: full	Hydrogen site location: inferred from neighbouring sites
*R*[*F*^2^ > 2σ(*F*^2^)] = 0.034	H-atom parameters constrained
*wR*(*F*^2^) = 0.091	*w* = 1/[σ^2^(*F*_o_^2^) + (0.045*P*)^2^ + 0.6567*P*] where *P* = (*F*_o_^2^ + 2*F*_c_^2^)/3
*S* = 1.10	(Δ/σ)_max_ = 0.001
3210 reflections	Δρ_max_ = 0.28 e Å^−^^3^
200 parameters	Δρ_min_ = −0.48 e Å^−^^3^
5 restraints	

### 2-Methoxy-4-(thiophen-2-yl)-5,6,7,8,9,10-hexahydro-cycloocta[b]pyridine-3-carbonitrile (1c) Special details

**Table:**

Geometry. All esds (except the esd in the dihedral angle between two l.s. planes) are estimated using the full covariance matrix. The cell esds are taken into account individually in the estimation of esds in distances, angles and torsion angles; correlations between esds in cell parameters are only used when they are defined by crystal symmetry. An approximate (isotropic) treatment of cell esds is used for estimating esds involving l.s. planes.

### 2-Methoxy-4-(thiophen-2-yl)-5,6,7,8,9,10-hexahydro-cycloocta[b]pyridine-3-carbonitrile (1c) Fractional atomic coordinates and isotropic or equivalent isotropic displacement parameters (Å^2^)

**Table:**

	*x*	*y*	*z*	*U*_iso_*/*U*_eq_	Occ. (<1)
N1	0.96546 (10)	0.70446 (8)	0.86883 (8)	0.0189 (2)	
C2	0.90244 (11)	0.79770 (9)	0.86327 (9)	0.0178 (2)	
C3	0.78613 (11)	0.82488 (9)	0.78055 (9)	0.0181 (2)	
C4	0.73796 (11)	0.75056 (9)	0.69719 (9)	0.0177 (2)	
C4A	0.80433 (12)	0.65081 (9)	0.70138 (9)	0.0182 (2)	
C5	0.75694 (12)	0.56848 (9)	0.61264 (10)	0.0212 (2)	
H5A	0.838085	0.525190	0.604922	0.025*	
H5B	0.721646	0.605961	0.542353	0.025*	
C6	0.64314 (13)	0.49310 (10)	0.63404 (10)	0.0258 (3)	
H6A	0.555045	0.533710	0.622776	0.031*	0.899 (3)
H6B	0.629649	0.435362	0.578674	0.031*	0.899 (3)
H6C	0.587821	0.464035	0.564337	0.031*	0.101 (3)
H6D	0.580013	0.531417	0.672098	0.031*	0.101 (3)
C7	0.67033 (14)	0.44193 (12)	0.74716 (12)	0.0266 (4)	0.899 (3)
H7A	0.591782	0.393868	0.750517	0.032*	0.899 (3)
H7B	0.672451	0.498915	0.802283	0.032*	0.899 (3)
C8	0.80578 (15)	0.37783 (11)	0.77828 (13)	0.0274 (4)	0.899 (3)
H8A	0.834831	0.358112	0.710285	0.033*	0.899 (3)
H8B	0.786865	0.310712	0.814129	0.033*	0.899 (3)
C9	0.92878 (13)	0.43620 (10)	0.85552 (11)	0.0277 (3)	
H9A	0.895112	0.465575	0.918164	0.033*	0.899 (3)
H9B	1.001371	0.382682	0.885016	0.033*	0.899 (3)
H9C	0.963248	0.367771	0.832114	0.033*	0.101 (3)
H9D	0.962418	0.440989	0.935936	0.033*	0.101 (3)
C7'	0.7263 (11)	0.3951 (8)	0.7125 (8)	0.018 (3)*	0.101 (3)
H7'1	0.663372	0.332962	0.709291	0.021*	0.101 (3)
H7'2	0.807401	0.372112	0.684096	0.021*	0.101 (3)
C8'	0.7754 (10)	0.4312 (9)	0.8312 (8)	0.021 (3)*	0.101 (3)
H8'1	0.736369	0.502322	0.841452	0.025*	0.101 (3)
H8'2	0.744341	0.379823	0.880831	0.025*	0.101 (3)
C10	0.99628 (12)	0.52748 (9)	0.80377 (10)	0.0215 (2)	
H10A	1.090157	0.540801	0.849794	0.026*	
H10B	1.007839	0.503787	0.731011	0.026*	
C10A	0.91682 (12)	0.63133 (9)	0.78995 (10)	0.0185 (2)	
O1	0.95029 (9)	0.87491 (6)	0.93712 (7)	0.02090 (19)	
C11	1.06586 (13)	0.84862 (10)	1.02549 (10)	0.0239 (3)	
H11A	1.144124	0.824411	0.995574	0.036*	
H11B	1.038666	0.791551	1.069970	0.036*	
H11C	1.093504	0.911983	1.071124	0.036*	
C12	0.72294 (12)	0.92762 (9)	0.78455 (9)	0.0196 (2)	
N2	0.67678 (11)	1.01077 (8)	0.79259 (9)	0.0247 (2)	
S1	0.66062 (3)	0.86051 (3)	0.50213 (3)	0.03155 (12)	
C13	0.62329 (12)	0.78155 (9)	0.60424 (9)	0.0195 (2)	
C14	0.48341 (13)	0.75720 (10)	0.58386 (10)	0.0250 (3)	
H14	0.441950	0.713919	0.630015	0.030*	
C15	0.40917 (14)	0.80518 (11)	0.48486 (12)	0.0320 (3)	
H15	0.311650	0.798012	0.458326	0.038*	
C16	0.49071 (15)	0.86180 (11)	0.43241 (12)	0.0330 (3)	
H16	0.457697	0.897968	0.364963	0.040*	

### 2-Methoxy-4-(thiophen-2-yl)-5,6,7,8,9,10-hexahydro-cycloocta[b]pyridine-3-carbonitrile (1c) Atomic displacement parameters (Å^2^)

**Table:**

	*U*^11^	*U*^22^	*U*^33^	*U*^12^	*U*^13^	*U*^23^
N1	0.0175 (4)	0.0169 (5)	0.0212 (5)	0.0006 (4)	0.0021 (4)	0.0021 (4)
C2	0.0176 (5)	0.0161 (5)	0.0195 (5)	−0.0015 (4)	0.0034 (4)	0.0008 (4)
C3	0.0164 (5)	0.0155 (5)	0.0219 (5)	0.0005 (4)	0.0032 (4)	0.0017 (4)
C4	0.0157 (5)	0.0178 (5)	0.0192 (5)	−0.0007 (4)	0.0029 (4)	0.0024 (4)
C4A	0.0182 (5)	0.0166 (5)	0.0197 (5)	−0.0002 (4)	0.0040 (4)	0.0010 (4)
C5	0.0228 (6)	0.0189 (5)	0.0205 (5)	0.0023 (4)	0.0019 (4)	−0.0010 (4)
C6	0.0250 (6)	0.0219 (6)	0.0271 (6)	−0.0029 (5)	−0.0016 (5)	−0.0001 (5)
C7	0.0208 (7)	0.0239 (7)	0.0341 (8)	0.0011 (5)	0.0044 (6)	0.0065 (6)
C8	0.0231 (7)	0.0196 (7)	0.0371 (8)	0.0012 (5)	0.0018 (6)	0.0058 (6)
C9	0.0236 (6)	0.0237 (6)	0.0335 (7)	0.0041 (5)	0.0015 (5)	0.0086 (5)
C10	0.0184 (5)	0.0183 (6)	0.0259 (6)	0.0030 (4)	0.0010 (4)	−0.0001 (4)
C10A	0.0173 (5)	0.0168 (5)	0.0213 (5)	0.0004 (4)	0.0043 (4)	0.0014 (4)
O1	0.0209 (4)	0.0169 (4)	0.0213 (4)	0.0014 (3)	−0.0028 (3)	−0.0013 (3)
C11	0.0239 (6)	0.0211 (6)	0.0221 (6)	0.0006 (5)	−0.0045 (5)	−0.0002 (4)
C12	0.0162 (5)	0.0200 (6)	0.0210 (5)	−0.0012 (4)	0.0003 (4)	0.0004 (4)
N2	0.0227 (5)	0.0208 (5)	0.0277 (5)	0.0027 (4)	−0.0002 (4)	−0.0010 (4)
S1	0.02601 (18)	0.0363 (2)	0.02987 (19)	−0.00005 (13)	0.00100 (13)	0.01445 (13)
C13	0.0201 (5)	0.0159 (5)	0.0209 (6)	0.0014 (4)	0.0014 (4)	0.0003 (4)
C14	0.0216 (6)	0.0233 (6)	0.0277 (6)	0.0017 (5)	0.0003 (5)	0.0048 (5)
C15	0.0235 (6)	0.0282 (7)	0.0374 (7)	0.0002 (5)	−0.0076 (5)	0.0022 (6)
C16	0.0340 (7)	0.0303 (7)	0.0286 (7)	0.0042 (5)	−0.0061 (5)	0.0075 (5)

### 2-Methoxy-4-(thiophen-2-yl)-5,6,7,8,9,10-hexahydro-cycloocta[b]pyridine-3-carbonitrile (1c) Geometric parameters (Å, º)

**Table:**

N1—C2	1.3168 (15)	C9—C10	1.5378 (17)
N1—C10A	1.3538 (15)	C9—H9A	0.9900
C2—O1	1.3482 (14)	C9—H9B	0.9900
C2—C3	1.4056 (15)	C9—H9C	0.9900
C3—C4	1.4003 (16)	C9—H9D	0.9900
C3—C12	1.4348 (16)	C7'—C8'	1.526 (12)
C4—C4A	1.4053 (16)	C7'—H7'1	0.9900
C4—C13	1.4826 (15)	C7'—H7'2	0.9900
C4A—C10A	1.4046 (16)	C8'—H8'1	0.9900
C4A—C5	1.5115 (16)	C8'—H8'2	0.9900
C5—C6	1.5365 (17)	C10—C10A	1.5078 (15)
C5—H5A	0.9900	C10—H10A	0.9900
C5—H5B	0.9900	C10—H10B	0.9900
C6—C7	1.5241 (19)	O1—C11	1.4391 (13)
C6—C7'	1.669 (10)	C11—H11A	0.9800
C6—H6A	0.9900	C11—H11B	0.9800
C6—H6B	0.9900	C11—H11C	0.9800
C6—H6C	0.9900	C12—N2	1.1494 (16)
C6—H6D	0.9900	S1—C16	1.7093 (14)
C7—C8	1.5333 (19)	S1—C13	1.7216 (12)
C7—H7A	0.9900	C13—C14	1.3815 (17)
C7—H7B	0.9900	C14—C15	1.4248 (18)
C8—C9	1.5552 (19)	C14—H14	0.9500
C8—H8A	0.9900	C15—C16	1.349 (2)
C8—H8B	0.9900	C15—H15	0.9500
C9—C8'	1.478 (10)	C16—H16	0.9500
			
C2—N1—C10A	118.31 (10)	H9A—C9—H9B	107.4
N1—C2—O1	120.66 (10)	C8'—C9—H9C	107.9
N1—C2—C3	123.49 (11)	C10—C9—H9C	107.9
O1—C2—C3	115.83 (10)	C8'—C9—H9D	107.9
C4—C3—C2	118.32 (10)	C10—C9—H9D	107.9
C4—C3—C12	122.93 (10)	H9C—C9—H9D	107.2
C2—C3—C12	118.74 (10)	C8'—C7'—C6	111.3 (8)
C3—C4—C4A	118.99 (10)	C8'—C7'—H7'1	109.4
C3—C4—C13	118.98 (10)	C6—C7'—H7'1	109.4
C4A—C4—C13	121.95 (10)	C8'—C7'—H7'2	109.4
C10A—C4A—C4	117.59 (10)	C6—C7'—H7'2	109.4
C10A—C4A—C5	121.53 (10)	H7'1—C7'—H7'2	108.0
C4—C4A—C5	120.87 (10)	C9—C8'—C7'	107.5 (8)
C4A—C5—C6	114.05 (10)	C9—C8'—H8'1	110.2
C4A—C5—H5A	108.7	C7'—C8'—H8'1	110.2
C6—C5—H5A	108.7	C9—C8'—H8'2	110.2
C4A—C5—H5B	108.7	C7'—C8'—H8'2	110.2
C6—C5—H5B	108.7	H8'1—C8'—H8'2	108.5
H5A—C5—H5B	107.6	C10A—C10—C9	115.14 (10)
C7—C6—C5	115.95 (10)	C10A—C10—H10A	108.5
C5—C6—C7'	105.8 (4)	C9—C10—H10A	108.5
C7—C6—H6A	108.3	C10A—C10—H10B	108.5
C5—C6—H6A	108.3	C9—C10—H10B	108.5
C7—C6—H6B	108.3	H10A—C10—H10B	107.5
C5—C6—H6B	108.3	N1—C10A—C4A	123.24 (10)
H6A—C6—H6B	107.4	N1—C10A—C10	114.11 (10)
C5—C6—H6C	110.6	C4A—C10A—C10	122.63 (10)
C7'—C6—H6C	110.6	C2—O1—C11	117.39 (9)
C5—C6—H6D	110.6	O1—C11—H11A	109.5
C7'—C6—H6D	110.6	O1—C11—H11B	109.5
H6C—C6—H6D	108.7	H11A—C11—H11B	109.5
C6—C7—C8	114.70 (12)	O1—C11—H11C	109.5
C6—C7—H7A	108.6	H11A—C11—H11C	109.5
C8—C7—H7A	108.6	H11B—C11—H11C	109.5
C6—C7—H7B	108.6	N2—C12—C3	176.64 (12)
C8—C7—H7B	108.6	C16—S1—C13	92.07 (6)
H7A—C7—H7B	107.6	C14—C13—C4	130.04 (11)
C7—C8—C9	115.32 (12)	C14—C13—S1	111.19 (9)
C7—C8—H8A	108.4	C4—C13—S1	118.77 (9)
C9—C8—H8A	108.4	C13—C14—C15	111.46 (12)
C7—C8—H8B	108.4	C13—C14—H14	124.3
C9—C8—H8B	108.4	C15—C14—H14	124.3
H8A—C8—H8B	107.5	C16—C15—C14	113.52 (12)
C8'—C9—C10	117.8 (5)	C16—C15—H15	123.2
C10—C9—C8	115.98 (11)	C14—C15—H15	123.2
C10—C9—H9A	108.3	C15—C16—S1	111.76 (10)
C8—C9—H9A	108.3	C15—C16—H16	124.1
C10—C9—H9B	108.3	S1—C16—H16	124.1
C8—C9—H9B	108.3		
			
C10A—N1—C2—O1	−177.20 (10)	C8'—C9—C10—C10A	33.5 (5)
C10A—N1—C2—C3	0.87 (17)	C8—C9—C10—C10A	78.19 (15)
N1—C2—C3—C4	−2.62 (17)	C2—N1—C10A—C4A	1.34 (17)
O1—C2—C3—C4	175.53 (10)	C2—N1—C10A—C10	179.91 (10)
N1—C2—C3—C12	177.99 (10)	C4—C4A—C10A—N1	−1.67 (17)
O1—C2—C3—C12	−3.86 (16)	C5—C4A—C10A—N1	177.53 (10)
C2—C3—C4—C4A	2.15 (16)	C4—C4A—C10A—C10	179.88 (10)
C12—C3—C4—C4A	−178.48 (11)	C5—C4A—C10A—C10	−0.92 (17)
C2—C3—C4—C13	−174.70 (10)	C9—C10—C10A—N1	99.52 (12)
C12—C3—C4—C13	4.66 (17)	C9—C10—C10A—C4A	−81.90 (15)
C3—C4—C4A—C10A	−0.16 (16)	N1—C2—O1—C11	−4.18 (16)
C13—C4—C4A—C10A	176.60 (10)	C3—C2—O1—C11	177.61 (10)
C3—C4—C4A—C5	−179.36 (10)	C3—C4—C13—C14	−101.27 (15)
C13—C4—C4A—C5	−2.61 (17)	C4A—C4—C13—C14	81.97 (17)
C10A—C4A—C5—C6	91.62 (13)	C3—C4—C13—S1	78.50 (13)
C4—C4A—C5—C6	−89.21 (13)	C4A—C4—C13—S1	−98.25 (12)
C4A—C5—C6—C7	−49.08 (15)	C16—S1—C13—C14	0.20 (10)
C4A—C5—C6—C7'	−85.2 (4)	C16—S1—C13—C4	−179.62 (10)
C5—C6—C7—C8	−56.74 (16)	C4—C13—C14—C15	179.12 (12)
C6—C7—C8—C9	100.76 (15)	S1—C13—C14—C15	−0.66 (14)
C7—C8—C9—C10	−72.30 (16)	C13—C14—C15—C16	0.95 (18)
C5—C6—C7'—C8'	77.4 (8)	C14—C15—C16—S1	−0.79 (17)
C10—C9—C8'—C7'	72.2 (8)	C13—S1—C16—C15	0.34 (12)
C6—C7'—C8'—C9	−111.6 (8)		

### 2-Methoxy-4-(thiophen-2-yl)-5,6,7,8,9,10-hexahydro-cycloocta[b]pyridine-3-carbonitrile (1c) Hydrogen-bond geometry (Å, º)

**Table:**

*D*—H···*A*	*D*—H	H···*A*	*D*···*A*	*D*—H···*A*
C11—H11*C*···N2^i^	0.98	2.69	3.4864 (16)	138
C16—H16···N2^ii^	0.95	2.41	3.3379 (17)	167

Symmetry codes: (i) −*x*+2, −*y*+2, −*z*+2; (ii) −*x*+1, −*y*+2, −*z*+1.
